# Tuning the exposure of BiVO_4_-{010} facets to enhance the N_2_ photofixation performance†

**DOI:** 10.1039/d1ra02739e

**Published:** 2021-08-27

**Authors:** Honghao Chu, Shisheng Zheng, Yang Li, Kuanda Xu, Qingshui Hong, Tangyi Li, Wenju Ren, Shunning Li, Zongwei Mei, Feng Pan

**Affiliations:** School of Advanced Materials, Peking University, Shenzhen Graduate School Shenzhen China meizw@pkusz.edu.cn panfeng@pkusz.edu.cn; Chongqing Key Laboratory of Chemical Process for Clean Energy and Resource Utilization, School of Chemistry and Engineering, Chongqing University Chongqing China; School of Advance Manufacturing Engineering, Chongqing University of Posts and Telcommunications Chongqing China; Chemistry and Chemical Engineering Guangdong Laboratory Shantou China

## Abstract

Effective separation of photoexcited carriers and chemisorption of the N_2_ molecule are two key issues to efficient nitrogen photofixation. The spatial charge separation of BiVO_4_ with anisotropic exposed facets, namely the transfer of photoexcited electrons and holes to {010} and {110} facets, respectively, helps to enhance the separation ability of photogenerated carriers. Theoretical calculation results predict that a surface oxygen vacancy is easier to form on the (010) facet than on the (110) facet of BiVO_4_. Accordingly, in this study, enhanced N_2_ photofixation performance has been achieved for the first time by tuning the exposure of {010} facets of BiVO_4_.

## Abstract 2

Effective separation of photoexcited carriers and chemisorption of the N_2_ molecule are two key issues to efficient nitrogen photofixation.

## Graphical abstract

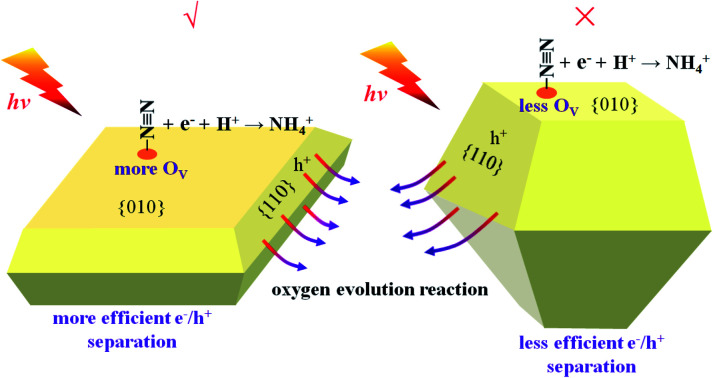

Nitrogen fixation to NH_3_ is an important artificial synthesis in the chemical industry.^1^ Nowadays, NH_3_ is commonly produced through the Haber–Bosch process, which requires high temperature (400–500 °C) and high pressure (15–25 MPa).^2^ This process accounts for ∼2% of the total global energy consumption and contributes ∼1.6% of the total global emissions.^3^ With the growing energy needs and demand for cleaner environment, a more environmentally friendly method is needed for NH_3_ manufacture. Photocatalytic N_2_ fixation, which employs solar energy and water to produce ammonium, is a promising sustainable and green strategy for NH_3_ synthesis compared with the traditional Haber–Bosch process.^4–9^ However, the photocatalytic performance of N_2_ fixation is far from satisfactory due to the inefficient separation of photogenerated carriers, the high activation energy barriers and hard cleavage of the strong N≡N triple bond energy (941 kJ mol^−1^) of the N_2_ molecule.^10^

Crystal facet engineering of semiconductors is a significant strategy for fine-tuning the charge separation of photocatalysts.^11^ Facet engineering of anatase TiO_2_ has been given considerable research attention for photocatalytic reaction by controlling the {001} exposure ratio.^12–14^ Recently, different research groups reported that the exposure of anisotropic facets of BiOX (X = Cl, Br, or I) enabled the directional transfer of photoexcited electrons and holes for spatial charge separation, accordingly improved the photocatalytic activities.^15–17^ Other semiconductors including SrTiO_3_,^18^ LaNbON_2_,^19^ and C_3_N_4_ (ref. 20) also demonstrated that the predominating anisotropic facet exposure could improve the separation of photogenerated carriers. Simultaneously, the spatial charge separation of BiVO_4_ with anisotropic exposed facets has been observed by direct imaging^21^ and photo-reduction or photo-oxidation reactions on {010} or {110} facets, respectively.^22–24^ These results indicated that the photoexcited electrons (e^−^) and holes (h^+^) would transfer to the {010} and {110} facets, respectively. Based on the special property of BiVO_4_, photocatalytic water splitting for O_2_ evolution has been greatly improved.^25^ However, a few researches on BiVO_4_ with anisotropic exposed facets have been reported for photocatalytic N_2_ fixation.

It is commonly understood that surface vacancies with abundant localized electrons play a critical role in N_2_ photofixation by capturing and activating the inert N_2_ molecule.^26,27^ Efficient transfer of the photogenerated electrons to the inert N_2_ molecule is also a key step for the effective photocatalytic N_2_ fixation.^28^ Considerable research results have revealed that surface oxygen vacancies could activate the N_2_ molecule by chemisorption, and act as the transfer bridge of photoexcited electrons from photocatalysts to the activated N_2_ molecule. For example, Pan *et al.* reported that the bond length of N≡N was elongated by the interaction with the surface O_VS_ on MoO_3−*x*_ nanobelts or W_18_O_49_ nanowires, and the photocatalytic activities for the N_2_ fixation were directly related to the surface O_VS_ concentration.^29,30^ The critical role of O_VS_ in the photofixation of N_2_ was also revealed by other semiconductors including TiO_2_,^10,31–33^ BiOCl,^34^ BiO quantum dots,^35^ ultrafine Cu_2_O,^8^ and amorphous CeO_*x*_.^36^ However, the surface O_VS_ on BiVO_4_ for N_2_ photofixation has been rarely studied.

Theoretical calculation results predicted that the formation energy (*E*_f_) of an O_V_ on the surface of the representative (010) facet was lower than that on the surface of the typical (110) facet, accordingly there were more O_VS_ on the surface of the (010) facet. Together with the spatial charge separation property, anisotropic exposed BiVO_4_ was believed to exhibit a good N_2_ photofixation performance. In this study, BiVO_4_ with anisotropic {010} and {110} facets were synthesized by a solid–liquid state reaction.^37^ It was first found that the as-prepared BiVO_4_ with higher percentage of exposed {010} facets exhibited better performance for N_2_ photofixation without any sacrificial reagent and cocatalyst under ambient conditions. The easier formation of a surface oxygen vacancy (O_V_) on the (010) facet was experimentally proved by the enhanced chemisorption of the N_2_ molecule based on the temperature programmed desorption (TPD) characterization. The enhanced separation of photogenerated carriers and more surface oxygen vacancies (O_VS_) made BiVO_4_ with a higher exposed ratio of {010} facets to be more efficient for photocatalytic N_2_ fixation.

The primitive unit cell consists of four units as shown by the side and top view of the optimized BiVO_4_ (Fig. 1a and b), and the optimized lattice parameters are as follows: *a* = 7.33 Å, *b* = 11.77 Å, *c* = 5.18 Å, and *β* = 134.92°. They are in good agreement with the experimental values: *a* = 7.25 Å, *b* = 11.70 Å, *c* = 5.09 Å, and *β* = 134.225°. The formation energy of the oxygen vacancy (*E*_f_) on the surface of (010) and (110) facets were calculated by following equation:*E*_f_ = *E*_O_V__ + *E*_½O_2__ − *E*_surface_where *E*_O_V__, *E*_½O_2__, and *E*_surface_ represent the total energy of surface with one oxygen vacancy, the half of total energy of oxygen and the total energy of surface without oxygen vacancy. The calculated formation energies of an oxygen vacancy were 2.91 eV and 4.93 eV on (010) and (110) surface, respectively. It means that the formation of a surface oxygen vacancy on the (010) facet was more energetically favorable than that on the (110) facet.

**Fig. 1:**
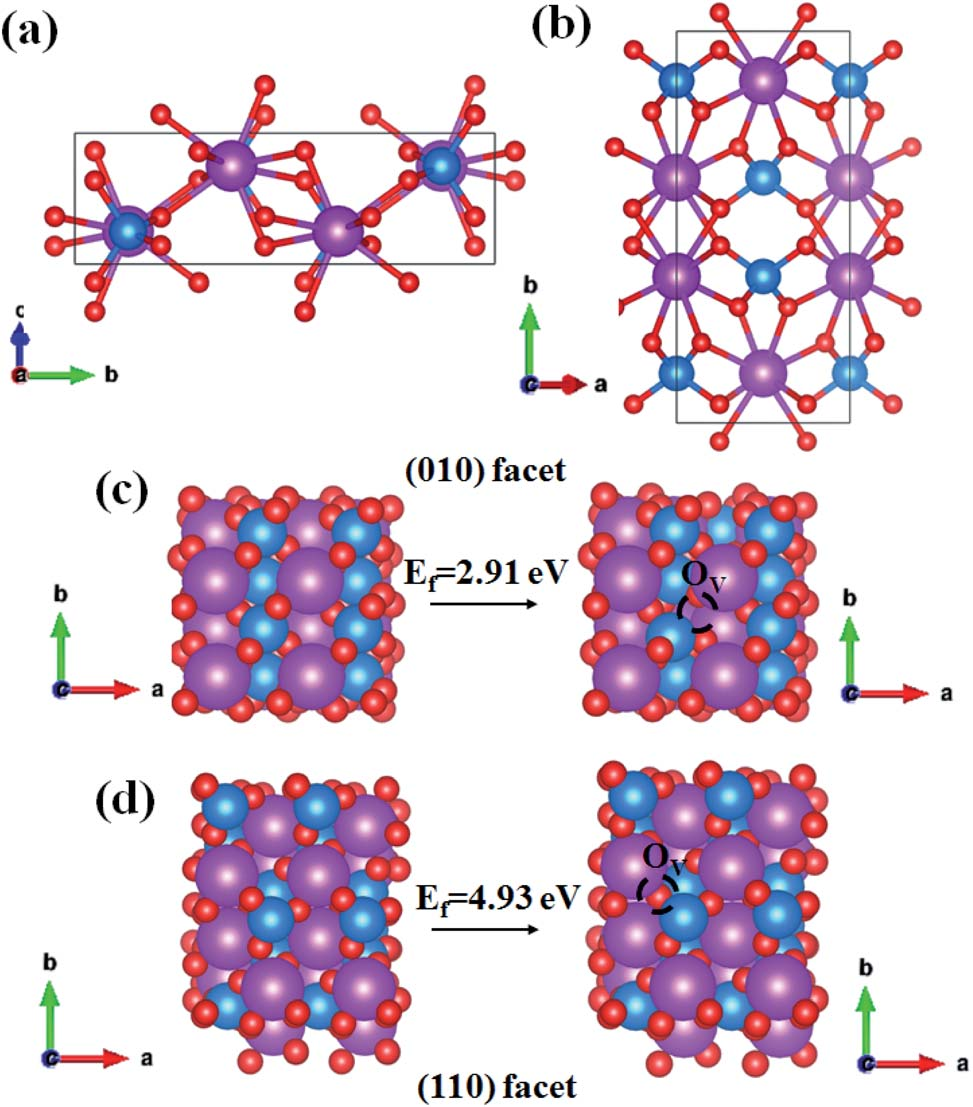
The side view (a) and top view (b) of optimized monoclinic BiVO_4_. The purple, blue and red atom indicates Bi, V and O, respectively; (c) the optimized structure of the (010) surface and (010) surface with one vacancy. (d) The optimized structure of the (110) surface and (110) surface with one vacancy.

Based on the theoretical calculation result, BiVO_4_ with different exposure ratios of {010} facets was fabricated by tuning the nitric concentration in the reaction solution according to a previous report (see ESI†). The as-synthesized BiVO_4_-0.50, BiVO_4_-0.75, and BiVO_4_-1.0 can be indexed to the monoclinic crystal structure (PDF# 14-0688) (Fig. 2a and S1†). However, the BiVO_4_-0.25 sample in Fig. S1† consists of a hybrid monoclinic structure (PDF# 14-0688) and a tetragonal crystal phase (PDF# 14-0133), probably due to the low concentration of the HNO_3_ solution. Furthermore, the (020) peak of BiVO_4_-0.50 appears around 15° of 2*θ* (Fig. 2a). The as-synthesized BiVO_4_ in different concentrations of HNO_3_ shows different facet exposure ratios of {010} to {110}, and BiVO_4_-0.50 exhibits the largest exposure ratio (Fig. 2b and S2†), which is consistent with the previous report.^37^ The largest exposure of {010} facets must cause the appearance of the (020) peak in Fig. 2a. The typical TEM image shows the regular shape of BiVO_4_-0.50 (Fig. 2c). The HRTEM interplanar spacing is 0.47 nm, corresponding to the value of (110) facet (Fig. 2d). The selected area electron diffraction (SAED) rings in the inset of Fig. 2d corresponds to the (110) and (040) crystal facets of monoclinic BiVO_4_.

**Fig. 2:**
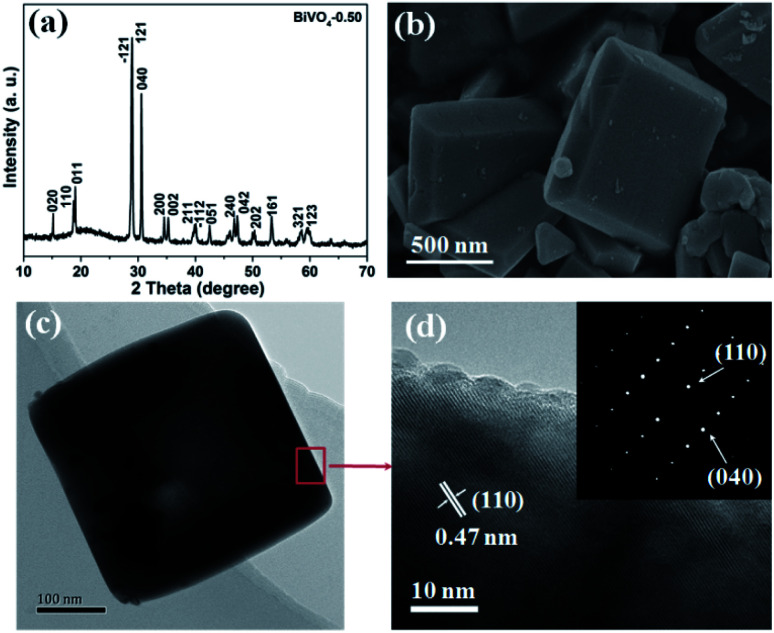
Typical XRD pattern (a), SEM image (b), TEM image (c), and HRTEM image (d) of the as-synthesized BiVO_4_-0.50. Inset in (d) SAED pattern.

Fig. 3 exhibits the temperature programmed desorption (TPD) characterization of the N_2_ molecule. A single peak centering at 220 °C is observed, which is ascribed to the desorption of the chemisorbed N_2_, and BiVO_4_-0.50 exhibits the strongest chemisorption of the N_2_ molecule. It is consistent with the theoretical calculation result shown in Fig. 1.

**Fig. 3:**
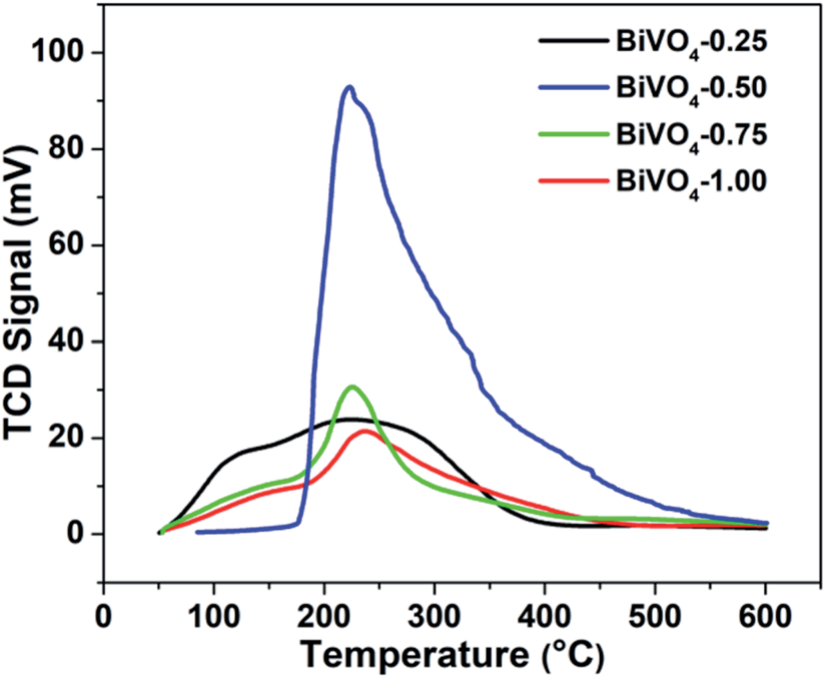
N_2_-TPD profiles of BiVO_4_ synthesized in the aqueous solution with different HNO_3_ concentrations.

For the general photocatalytic N_2_ photofixation process in pure water, the photoexcited electrons are injected into the chemisorbed N_2_ molecule *via* oxygen vacancy, and then the activated N_2_ molecule combines with H^+^ from water to form NH_3_. Simultaneously, the photogenerated holes oxidize the OH^−^ from water to produce O_2_. The standard curve from the different concentrations of NH_4_^+^ is shown in Fig. S4.† Photocatalytic performance test indicates that BiVO_4_-0.50 exhibits the best activity for N_2_ fixation among these four samples (Fig. 4a). The average NH_4_^+^ evolution rate is about 15.5 μmol g^−1^ L^−1^ h^−1^ for BiVO_4_-0.50 during a 6 h test. The average NH_4_^+^ evolution rates decrease to 13.1, 5.35, and 5.3 μmol g^−1^ L^−1^ h^−1^ for BiVO_4_-0.75, BiVO_4_-1.00, and BiVO_4_-0.25, respectively (Fig. 4a). The Brunauer–Emmett–Teller (BET) measurements indicate that the surface areas are 2.9 m^2^ g^−1^, 2.0 m^2^ g^−1^, 1.8 m^2^ g^−1^, and 1.5 m^2^ g^−1^ for BiVO_4_-0.25, BiVO_4_-0.50, BiVO_4_-0.75, and BiVO_4_-1.00, respectively. The corresponding ratios of the average NH_4_^+^ evolution rate to surface area are 4.5 μmol L^−1^ h^−1^ m^−2^, 7.8 μmol L^−1^ h^−1^ m^−2^, 3.0 μmol L^−1^ h^−1^ m^−2^, and 3.5 μmol L^−1^ h^−1^ m^−2^. It is obvious that the surface area is not the determining factor of the photocatalytic activity. In order to confirm the origination of the nitrogen element in NH_4_^+^, Ar gas was bubbled and the aqueous suspension of the BiVO_4_-0.50 sample was irradiated by a 300 W xenon lamp during the test time. It is found that there is no detectable NH_4_^+^ (Fig. 4a), and it can be concluded that the detected NH_4_^+^ did not originate from environmental contamination, but from the photofixation reaction of N_2_ molecule by the BiVO_4_ photocatalyst. Fig. 4b shows the light-wavelength-dependent photofixation activity of BiVO_4_-0.50. The photocatalytic performance decreases from 365 nm and exhibits almost no NH_4_^+^ from 515 nm, and no NH_4_^+^ is detected at 590 nm due to the light absorption range (Fig. S3†). The photocatalyst possessed photoexcited electrons with higher energy under the irradiation of the shorter wavelength light, and the photofixation of N_2_ was more easy to occur. However, the photofixation reaction of N_2_ molecule could not occur when the longer wavelength light was unable to excite the BiVO_4_ photocatalyst. This result further confirmed the photofixation of N_2_ in our study. The calculated value of quantum efficiency (QE) at 365 nm was about 0.003%.

**Fig. 4:**
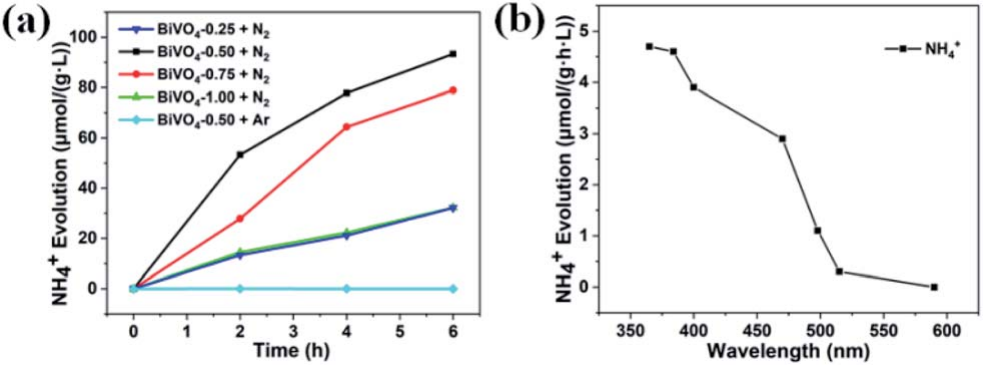
(a) Photocatalytic nitrogen fixation performance of BiVO_4_ samples synthesized with different concentrations of nitric acid (light source: 300 W xenon lamp; photocatalyst: 0.05 g; reaction solution: 100 ml of pure water). (b) Nitrogen fixation performance of BiVO_4_-0.50 illuminated by LED with different wavelengths (365 nm, 384 nm, 400 nm, 470 nm, 498 nm, 515 nm, and 590 nm).

In order to evaluate the stability of BiVO_4_-0.50 for N_2_ photofixation, three cycle tests were carried out, as exhibited in Fig. 5. After each cycle, the photocatalyst was washed by vacuum filtration with pure water for several times and dried in a vacuum oven. The photocatalytic activity gradually decreased probably due to the loss of photocatalyst during the wash process.

**Fig. 5:**
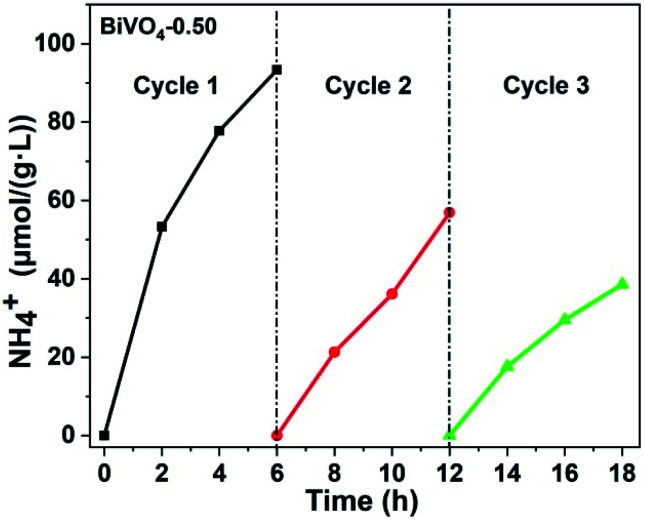
Photocatalytic stability test for BiVO_4_-0.50 (light source: 300 W xenon lamp; photocatalyst: 0.05 g; reaction solution: 100 ml of pure water).

In summary, BiVO_4_ with exposed anisotropic {010} and {110} facets was synthesized, and studied for the first time for ammonia production by photocatalytic N_2_ fixation from pure water. It was found that the sample with the high facet exposure ratio of {010} to {110} exhibited a better photocatalytic performance for N_2_ fixation, which resulted from the better separation ability of the photoexcited carriers and more surface O_VS_ on the {010} facet. The photoexcited electrons more effectively transferred to the more surface O_VS_ on the typical (010) facet, where the N_2_ molecule can be activated, and accordingly benefited to the enhancement of the photocatalytic performance. Our study provides a good strategy to improve the photocatalytic activity for N_2_ fixation.

## Conflicts of interest

There are no conflicts to declare.

## Supplementary Material

RA-011-D1RA02739E-s001
