# Supplementary material for: Tuning the exposure of BiVO4-{010} facets to enhance the N2 photofixation performance
Source: RSC Adv. 2021 Aug 27;11(46):28908–11. doi: 10.1039/d1ra02739e (PMC9038164; doi:10.1039/d1ra02739e)
Supplement: RA-011-D1RA02739E-s001 [file RA-011-D1RA02739E-s001.pdf]

Electronic Supplementary Material (ESI) for RSC Advances.
This journal is © The Royal Society of Chemistry 2021

Supplementary Information

Tuning the exposure of $BiVO_4$-{010} facets to enhance the N_2 photofixation performance

Honghao Chu[1], Shisheng Zheng[1], Yang Li[1], Kuanda Xu[1], Qingshui Hong[2], Tangyi Li[1], Wenju Ren[3], Shunning Li[1], Zongwei Mei*[1], Feng Pan*[1,4]

Affiliations:

[1]School of Advanced Materials, Peking University, Shenzhen Graduate School, Shenzhen, China.

[2]Chongqing Key Laboratory of Chemical Process for Clean Energy and Resource Utilization, School of Chemistry and Engineering, Chongqing University, Chongqing, China.

[3]School of Advance Manufacturing Engineering, Chongqing University of Posts and Telcommunications, Chongqing, China.

[4]Chemistry and Chemical Engineering Guangdong Laboratory, Shantou, China.

*Correspondence to: meizw@pkusz.edu.cn, panfeng@pkusz.edu.cn

Experimental Section

1. Synthesis of $BiVO_4$

In a typical procedure, 2.5 mmol of bismuth oxide (Bi_2O_3, 99.9%) and 2.5 mmol of vanadium oxide (V_2O_5, 99.7%) were mixed into 25 mL of nitric acid (HNO_3) with four different concentrations (0.25 M, 0.5 M, 0.75 M, and 1.0 M). Then the mixture was magnetically stirred for 4 days at room temperature. The yellow product was collected and washed with distilled water by the vacuum filtration route, and finally dried at 110 °C for further characterizations. Hereinafter the samples were displayed as $BiVO_4$-0.25, $BiVO_4$-0.5, $BiVO_4$-0.75, and $BiVO_4$-1.0 corresponding to the nitric acid concentration.

2. Photocatalytic test for N_2 fixation

The nitrogen photofixation performance was carried out in a double-walled quartz reactor. 50 mg of the as-synthesized $BiVO_4$ photocatalyst was added into 100 mL of deionized water to form a suspension that was dispersed by ultrasonication for 10 min. Then the suspension was irradiated by a 300 W Xe lamp (CEL-HXF300) after 0.5 h of nitrogen bubbling under mild stirring. The reactor temperature was held at 25 °C using a water thermostat. During the photocatalytic reaction process, 3 mL of the suspension was collected and immediately centrifuged to separate the dissolved products from the solid catalyst. The concentration

of NH_4^+ in the solution was measured by an ion chromatography (Metrohm Eco IC). And all the concentration values were based on the standard curve from different concentration of NH_4^+.

To evaluate the quantum efficiency (QE) of the as-synthesized $BiVO_4$ photocatalyst, LED lights with different wavelengths (365 nm, 384 nm, 400 nm, 470 nm, 498 nm, 515 nm, and 590 nm) were used as the light source. The light intensity was tested by PM100D with S401C (Thorlabs, USA). The QE value of ammonia was calculated based on the following equations.

$$QE_{ammonia}(\%) = 100 \times \frac{number\ of\ molcules\ ammonia\ \times 3}{number\ of\ incident\ photons} = \frac{100 \times \text{[illegible]}}{P \times \text{[illegible]}}$$

where *M* represents the amount of ammonia, N_A is the Avogadro's constant, *P* is the power of the incident light, λ is light irradiation wavelength, *h* is Plank constant, *c* is the light speed, and *t* is the light irradiation time.

3. Characterization of the photocatalysts

X-ray diffraction (XRD, Bruker, D8 Advance X-ray diffractometer) was used to characterize the crystal structure of the samples with the scanning range of 2θ from 10° to 70°. The morphology of the samples was observed on a field emission scanning electron microscope (ZEISS

SUPRA®55). Transmission electron microscope (TEM) imaging and high resolution-transmission electron microscopy (HRTEM) were performed on a Double-Cs aberration-corrected Themis G2 microscope at 300 kV with a Quantum GIF detector. UV-vis absorption spectroscopy was carried out on a UV-2450 (Shimadzu). The specific surface areas of the samples were determined through N_2 adsorption–desorption isotherms at 77 K using an accelerated surface area and porosimetry system (ASAP, 2020 HD88). The temperature-program desorption of N_2 (N_2-TPD) was tested with an Autosorb-iQ-C chemisorption analyzer (Quantachrome, USA) with the temperature from 50 to 600 °C.

4. DFT calculations

All the calculations were performed by means of spin-polarized DFT + U using Vienna ab initio simulation pack (VASP) with projector augmented wave (PAW) method. A plane-wave cutoff energy of 520 eV was adopted and the Perdew–Burke–Ernzerhof (PBE) exchange-correlation functional was employed. To complement the deficiencies of DFT in dealing with dispersion interactions, DFT–D3 semi-empirical Vander Waals corrections were included for structural optimization. AU value of 2.7 eV was applied for the V 3d states. A vacuum space of at least 15 Å was used to separate the surface slabs. The Brillouin zone was sampled by Γ-centered Monk horst–Pack (MP) k-point mesh of 5 × 3 × 7 for the

primitive cell relaxation.

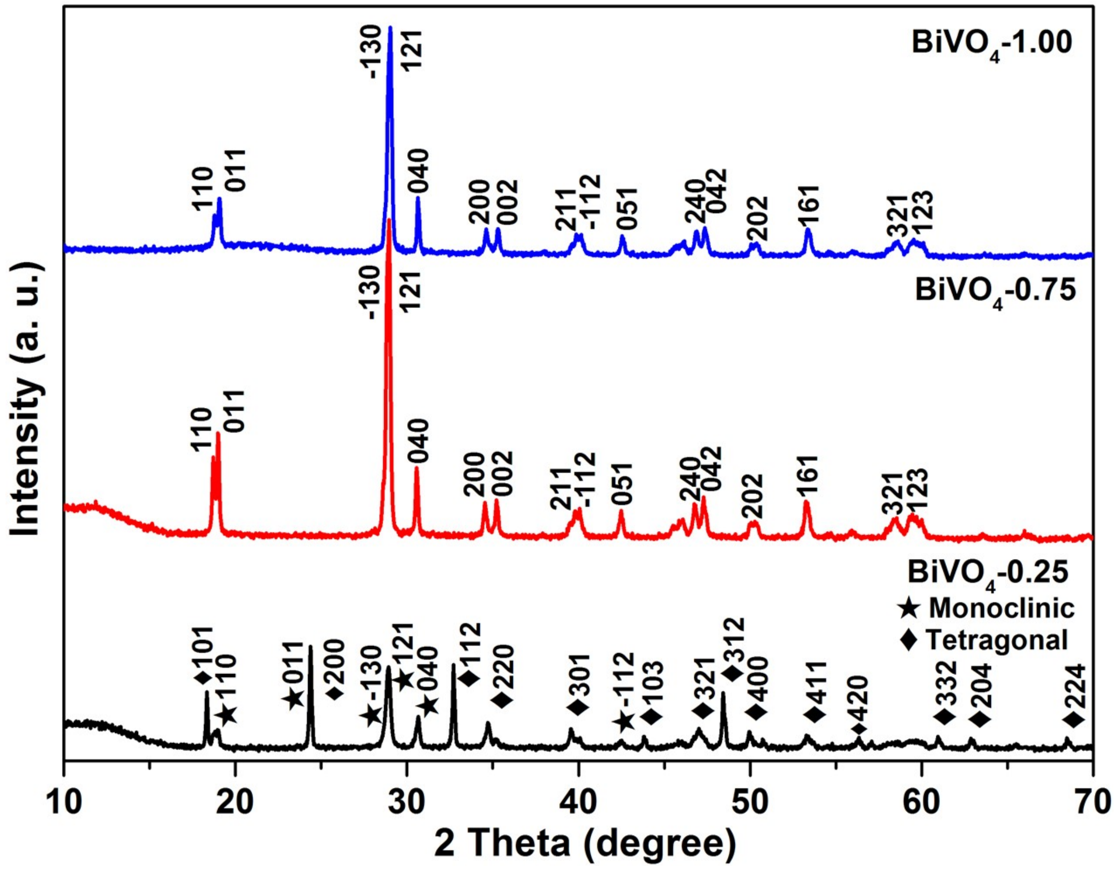

Fig. S1 Typical XRD patterns of as-synthesized $BiVO_4$-0.25, $BiVO_4$-0.75, and $BiVO_4$-1.00.

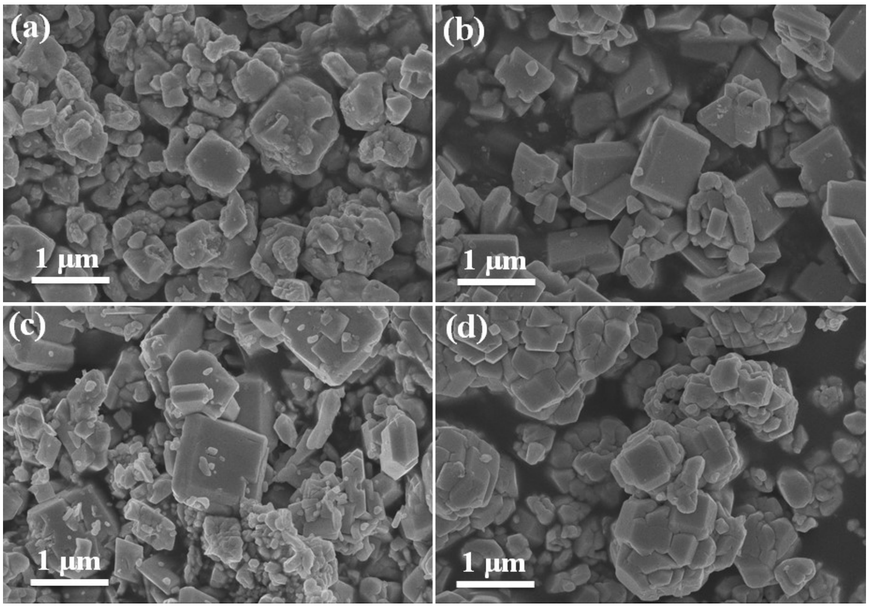

Fig. S2 Typical SEM images of (a) $BiVO_4$-0.25, (b) $BiVO_4$-0.50, (c) $BiVO_4$-0.75, and (d) $BiVO_4$-1.00.

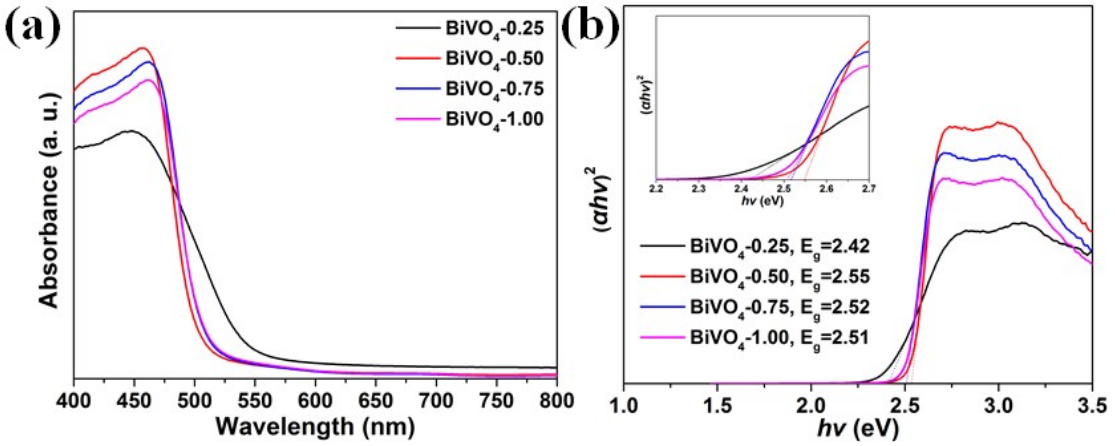

Fig. S3 (a) UV－Vis absorption spectra transformed from diffuse reflectance spectra, and (b) $(\alpha hv)^2 \sim hv$ plots of $BiVO_4$-0.25, $BiVO_4$-0.50, $BiVO_4$-0.75, and $BiVO_4$-1.00 samples. Inset in (b): the enlarged picture from 2.3 to 2.7 eV of $(\alpha hv)^2 \sim hv$ plots.

Shown in Fig. S3a is the typical UV-vis DRS spectra of the as-synthesized $BiVO_4$-0.25, $BiVO_4$-0.50, $BiVO_4$-0.75, $BiVO_4$-1.00. All the samples exhibit strong absorption in the visible range around 500 nm. And band gaps of the as-synthesized samples can be experimentally calculated based on the Kubelka-Munk (KM) method through the UV-vis absorption spectra transformed from the reflectance spectra by the following equation:

$$\alpha hv = A(hv - Eg)^{1/2}$$

where α is the absorption coefficient of UV-vis characterization, hv is the photon energy, Eg is the direct band gap, and A is a constant. Fig. S3b is the relational graph of $(\alpha hv)^2 \sim hv$ based on the above equation, from which we can calculate the band gap of the $BiVO_4$ synthesized in HNO_3

with different concentration. The values are 2.48, 2.53, 2.50, and 2.49 eV for $BiVO_4$-0.25, $BiVO_4$-0.50, $BiVO_4$-0.75, and $BiVO_4$-1.00, respectively.

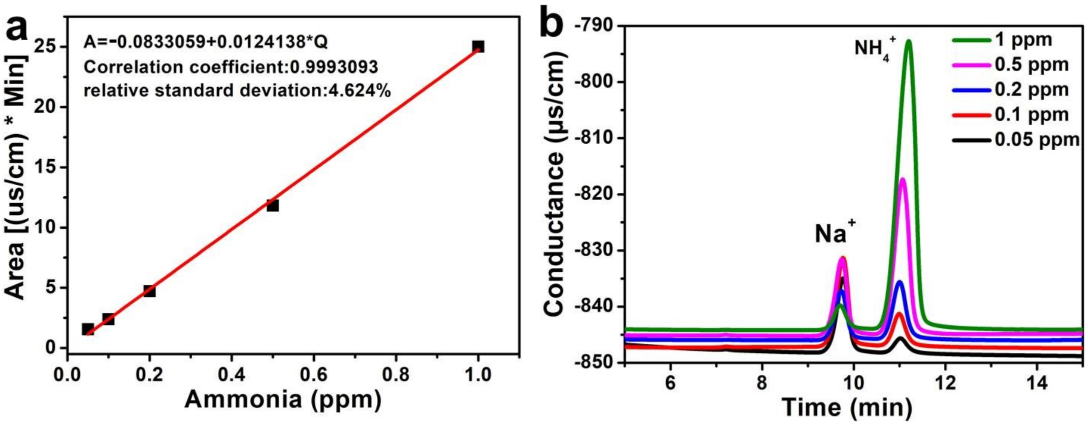

Fig. S4 (a) Standard curve of ammonia with high-performance ion chromatography. (b) Measurement curves of NH_4^+ from 0.05 ppm to 1 ppm tested by the ion chromatography.
